# Predictive Factors for Recurrence of Papillary Thyroid Carcinoma in Children and Adolescents

**DOI:** 10.3389/fonc.2022.833775

**Published:** 2022-02-24

**Authors:** Yan Gui, Dongmei Huang, Yun Hou, Xudong Wei, Jinming Zhang, Junyi Wang

**Affiliations:** ^1^ The First Hospital of Lanzhou University, Department of Otorhinolaryngology Head and Neck Surgery, Lanzhou City, China; ^2^ Department of Thyroid and Neck Tumor, Tianjin Medical University Cancer Institute and Hospital, National Clinical Research Center for Cancer, Key Laboratory of Cancer Prevention and Therapy, Tianjin’s Clinical Research Center for Cancer, Tianjin, China; ^3^ Department of Ear Nose Throat (E.N.T.), Gansu Provincial Hospital, Lanzhou, China; ^4^ The First School of Clinical Medicine, Lanzhou University, Lanzhou, China; ^5^ The First School of Clinical Medicine, Gansu University of Chinese Medicine, Lanzhou, China

**Keywords:** papillary thyroid carcinoma, children and adolescents, lymph node metastases, recurrence, prognosis

## Abstract

**Background:**

The incidence of papillary thyroid carcinoma (PTC) in children and adolescents has increased, but the data on long-term outcomes are limited. There are few literatures on the clinicopathological characteristics and prognosis of PTC in children and adolescents in China. Therefore, it is necessary to identify clinicopathological features to precisely predict clinical prognosis and to help choose the optimal method and perform the best therapeutic regimen.

**Methods:**

This study was a retrospective analysis of patients undergoing thyroidectomy at Tianjin Medical University Cancer Institute and Hospital. We analyzed the factors related to the clinicopathological features and prognosis of PTC in children and adolescents.

**Results:**

A total of 95 juvenile PTC patients who underwent thyroidectomy were enrolled. Our research found that patients with younger age (<14 years) were predominantly multifocal and have positive preoperative thyroglobulin (Tg) and higher recurrence rate, and their number of lymph node metastases (LNMs) was more than that of the older group (14–18 years). Maximal tumor size >2 cm, T stage, and multifocality were the risk factors for LNM and the number of LNM (p < 0.05). Multivariate analysis displayed the number of central LNM as the independent risk factor for lateral LNM, and multifocality was the independent risk factor for the number of central and lateral LNM. Younger age at diagnosis, positive preoperative thyroid-stimulating hormone (TSH), maximal tumor size >2 cm, lateral LNM, number of LNM, N staging, and American Thyroid Association (ATA) pediatric risk were related to poor prognosis in PTC patients (p < 0.05). Cox regression analysis found that younger age at diagnosis and positive preoperative TSH were independent risk factors for recurrence of PTC in children and adolescents.

**Conclusions:**

Our study showed that the clinicopathological characteristics of younger age compared with older age were as follows: highly aggressive, prone to metastases, and higher recurrence rate. In our opinion, patients with characteristics such as younger age at diagnosis, positive preoperative TSH, maximal tumor size >2 cm, lateral LNM, and number of LNM >5 may be considered for prophylactic or therapeutic dissection of additional metastatic LNs by high-volume surgeons to prevent and reduce the recurrence rate of patients during long-term follow-up.

## Introduction

Thyroid carcinoma is rare in children and adolescents, but the occurrence has been steadily rising worldwide in the past decades (1, 2). Papillary thyroid carcinoma (PTC) is the most common type of thyroid cancer in children and adolescents, which accounts for 90% (3, 4). Although the prognosis of pediatric patients with PTC is excellent and the mortality rate is low, they have higher rates of cervical lymph node metastases (LNMs), extrathyroidal extension (ETE), distant metastasis, and recurrence than the adults (5–8). Furthermore, a second operation for relapsed patients has a great impact on the quality of life of children and adolescents with PTC. In this setting, optimal treatment strategies for children and adolescents with PTC remain controversial. Therefore, recognizing the risk of recurrence of each patient may avoid an ineffective cure. PTC in children and adolescents has an increasing incidence, but long-term prognosis data are limited. Thence, recording the prognosis and identifying the predictors of PTC recurrence are of great clinical value in this age group.

There are few literatures on the clinicopathological characteristics and prognosis in children and adolescents with PTC in China. Therefore, identifying clinicopathological features to predict clinical prognosis and to help choose the optimal method and perform the best therapeutic regimen is crucial. For this reason, we carried out this study to find out the clinicopathological features and clinical outcomes of PTC in children and adolescents in China. According to the recent American Thyroid Association (ATA) guidelines (3), patients below 18 years with PTC were included in our study.

## Methods

### Study Population

This retrospective study was conducted at a single center institution; 95 patients who underwent initial thyroidectomy at Tianjin Medical University Cancer Hospital were recruited from January 2000 to August 2018. All patients met the following criteria: 1) they have confirmed PTC after thyroidectomy; 2) their age at diagnosis ≤18 years; 3) their tumors did not merge with other tumors; 4) they did not have a history of thyroidectomy or radiotherapy of the head and neck region; and 5) their medical records were complete. This study was approved by the Ethical Committee of the Tianjin Medical University Cancer Institute and Hospital.

### Clinicopathological Variables

Patient characteristics such as age at diagnosis, gender, serological test [e.g., thyroid peroxidase antibody (TPOAb), thyroglobulin antibody (TgAb), thyroid-stimulating hormone (TSH), and thyroglobulin (Tg)], surgery approach, lymph node dissection, pathological characteristics of maximal tumor size, bilaterality, ETE, multifocality, LNM and the number of LNM, and postoperative histological type were recorded completely. The normal ranges of TPOAb, TgAb, TSH, and Tg were 0–9 IU/ml, 0–4.1 IU/ml, 0.27–4.20 mlU/L, and 1.4–78 μg/L, respectively. The histological diagnoses were confirmed by 2 independent pathologists at our institution. TPOAb, TgAb, TSH, and Tg were considered positive when its result was over the upper range. Multifocality was considered if there are two or more tumor foci within the thyroid. Bilaterality was defined if tumors were located in both lobes. The ATA initial risk stratification (3) was performed considering the characteristics of recurrent tumors. Low risk was defined as disease grossly confined to the thyroid with N0/Nx disease or patients with incidental N1a disease (microscopic metastasis to a small number of central neck lymph nodes). Intermediate risk was defined as extensive N1a or minimal N1b disease, and high risk was defined as a regionally extensive disease (extensive N1b) or locally invasive disease (T4 tumors), with or without distant metastasis.

### Postoperative Follow-Up

The primary outcome was recurrence of disease in our study, which was assessed from records of basal or stimulated Tg, postoperative neck ultrasonography, ^131^I whole-body scans, and LN biopsies and pathologically diagnosed after the second operation. Disease-free survival (DFS) is defined as the time interval from thyroidectomy to detection of recurrent PTC. The follow-up period of each patient was defined as the length of time from the initial therapy to the last known contact, which was recorded by viewing the medical history or calling the patient.

### Statistical Analysis

Statistical analysis was performed by using the SPSS v26.0 (Chicago, IL, USA). Results of continuous variables were reported as mean ± SD or median values and ranges, and categorical variables were reported as absolute numbers and percentages. Intergroup differences were assessed with the independent-samples t-test or the Mann–Whitney U test (continuous variables) and the χ^2^-test with Yates’s correction or Fisher’s exact test (categorical variables), as appropriate. Recurrence-free survival plots were constructed by using the Kaplan–Meier method, and groups were compared by using log-rank tests. The Cox hazards regression model was used in multivariate analysis; the hazard ratio (HR) with the 95% CI was presented. A value of p < 0.05 was considered statistically significant. All data were analyzed anonymously.

## Results

### Study Populations

A total of 95 PTC patients, children and adolescents, who underwent thyroidectomy were recruited. The features of patients are given in **Table 1**. The study patients consisted of 64 girls (67.4%) and 31 boys (32.6%) with a median age of 14 years (range: 5–18 years). Thyroid involvement was multifocal in 57 patients (60.0%) and bilateral in 33 patients (34.7%). ETE was documented in the tumors of 67 patients (70.5%). A total of 18 patients underwent unilateral central lymph node dissection (CLND), 28 patients underwent unilateral modified radical neck dissection (MRND), 12 patients underwent unilateral MRND plus contralateral CLND, 6 patients underwent bilateral CLND, and 31 patients underwent bilateral MRND. A total of 84 had central LNM (88.4%), and 66 had lateral LNM (69.5%). The median (range) number of total LN dissected was 39 (0–136), and total LNM was 11 (0–44). During a mean follow-up of 63 months (2–193 months), 26.3% of patients were classified into low-risk groups, 24.2% into intermediate-risk groups, and 49.5% into high-risk groups according to the ATA pediatric risk stratification. A total of 29 patients (30.5%) had a recurrence.

**Table 1:** Characteristics of the study patients.

Characteristics	Value	Characteristics	Value
Age at diagnosis		Bilaterality	
<14 years	36 (37.9)	Yes	33 (34.7)
14–18 years	59 (62.1)	No	62 (65.3)
Gender		Central LNM	
Female	64 (67.4)	Yes	84 (88.4)
Male	31 (32.6)	No	11 (11.6)
Preoperative TPOAb level		Lateral LNM	
Positive	29 (30.5)	Yes	66 (69.5)
Negative	66 (69.5)	No	29 (30.5)
Preoperative TgAb level		N stage	
Positive	25 (26.3)	N0	6 (6.3)
Negative	70 (73.7)	N1a	23 (24.2)
Preoperative TSH level		N1b	66 (69.5)
Positive	19 (20.0)	Surgical approach	
Negative	76 (80.0)	Total bilateral thyroidectomy	41 (43.2)
Preoperative Tg level		Ipsilateral glandular lobe plus isthmus resection	42 (44.2)
Positive	34 (35.8)	Subtotal thyroidectomy	12 (12.6)
Negative	61 (64.2)	Lymph node dissection	
Maximal tumor size		Unilateral CLND	18 (18.9)
≤2 cm	40 (42.1)	Unilateral MRND	28 (29.5)
>2 cm	55 (57.9)	Unilateral MRND, plus contralateral CLND	12 (12.6)
T stage		Bilateral CLND	6 (6.3)
T1a	5 (5.3)	Bilateral MRND	31 (32.6)
T1b	35 (36.8)	No. of total LND	39 (0-136)
T2	49 (51.6)	No. of total LNM	11 (0-44)
T3	6 (6.3)	ATA pediatric risk	
Multifocality		Low	25 (26.3)
Yes	57 (60.0)	Intermediate	23 (24.2)
No	38 (40.0)	High	47 (49.5)
Extrathyroidal extension		Recurrence	
Yes	67 (70.5)	Yes	29 (30.5)
No	28 (29.5)	No	66 (69.5)

TPOAb, thyroid peroxidase antibody; TgAb, thyroglobulin antibody; TSH, thyroid-stimulating hormone; Tg, thyroglobulin; LNM, lymph node metastases; CLND, central lymph node dissection; MRND, modified radical neck dissection; LND, lymph node dissection; ATA, American Thyroid Association.

### Comparison Between Clinicopathological Features With Different Age Groups in Children and Adolescents With Papillary Thyroid Carcinoma

**Figure 1:**
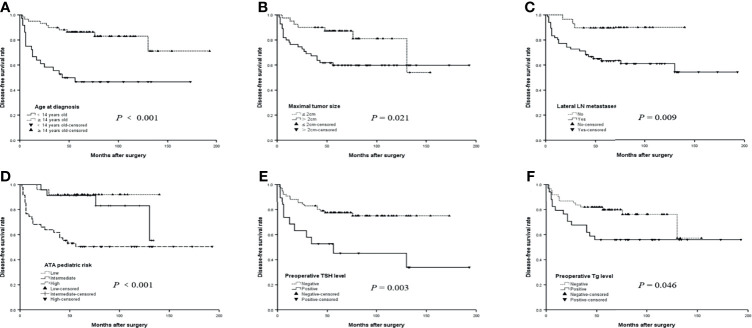
The disease-free survival (DFS) curves of risk factors for recurrence in children and adolescents with papillary thyroid carcinoma (PTC). **(A)** The DFS curves of age at diagnosis in children and adolescents with PTC. **(B)** The DFS curves of maximal tumor size in children and adolescents with PTC. **(C)** The DFS curves of lateral LN metastases in children and adolescents with PTC. **(D)** The DFS curves of ATA pediatric in children and adolescents with PTC. **(E)** The DFS curves of Preoperative TSH level in children and adolescents with PTC. **(F)** The DFS curves of Preoperative Tg level in children and adolescents with PTC.

We compared the clinicopathological features between different age groups (<14 and 14–18 years). We observed that there were statistically significant differences between multifocality, preoperative TSH level, surgical approach, LN dissection, the number of LND and LNM, and recurrence (**Table 2**). Our research showed that patients in the younger group (<14 years) were predominantly multifocal (p = 0.020), their preoperative Tg was often abnormal (p = 0.032), and they had more LND and LNM and higher recurrence rate than the older group (14–18 years) (p < 0.05). Moreover, the DFS rate was significantly different between different age groups (p < 0.001, **Figure 1A**).

**Table 2:** Comparison of clinicopathological features of PTC in children and adolescents with different age groups.

Variables	<14 years (n = 36)	14–18 years (n = 59)	p-Value
Gender			
Male	12 (33.3)	19 (32.2)	0.909
Female	24 (66.7)	40 (67.8)	
Maximal tumor size			
≤2 cm	11 (30.6)	29 (49.2)	0.075
>2 cm	25 (69.4)	30 (50.8)	
T stage			
T1a	0 (0.0)	5 (8.5)	0.167
T1b	11 (30.6)	24 (40.7)	
T2	22 (61.1)	27 (45.8)	
T3	3 (8.3)	3 (5.1)	
Central LNM			
Yes	35 (97.2)	49 (83.1)	0.078
No	1 (2.8)	10 (16.9)	
Lateral LNM			
Yes	29 (80.6)	37 (62.7)	0.067
No	7 (19.4)	22 (37.2)	
N stage			
N0	1 (2.8)	5 (8.5)	0.209
N1a	6 (16.7)	17 (28.8)	
N1b	29 (80.6)	37 (62.7)	
Multifocality			
Yes	27 (75.0)	30 (50.8)	0.020
No	9 (25.0)	29 (49.2)	
Extrathyroidal extension			
Yes	25 (69.4)	42 (71.2)	0.857
No	11 (30.6)	17 (28.8)	
Bilaterality			
Yes	16 (44.4)	42 (71.2)	0.121
No	20 (55.6)	17 (28.8)	
Preoperative TSH level	6.6 ± 16.5	2.9 ± 2.1	0.032
Preoperative Tg level	125.3 ± 203.5	129.3 ± 166.9	0.419
Surgical approach			
Total bilateral thyroidectomy	23 (63.9)	18 (30.5)	<0.001
Ipsilateral glandular lobe plus isthmus resection	7 (19.4)	35 (59.3)	
Subtotal thyroidectomy	6 (16.7)	6 (10.2)	
Lymph node dissection			
Unilateral CLND	4 (11.1)	14 (23.7)	0.003
Unilateral MRND	7 (19.4)	21 (35.6)	
Unilateral MRND, plus contralateral CLND	2 (5.6)	10 (16.9)	
Bilateral CLND	3 (8.3)	3 (5.1)	
Bilateral MRND	20 (55.6)	11 (18.6)	
Number of central LND	12.6 ± 6.8	7.9 ± 5.2	<0.001
Number of lateral LND	45.9 ± 33.8	26.2 ± 25.3	0.002
Number of LND	58.5 ± 37.0	34.1 ± 27.3	0.001
Number of central LN metastases	7.1 ± 4.3	4.6 ± 3.8	0.005
Number of lateral LN metastases	10.7 ± 8.6	5.3 ± 6.7	0.002
Number of LN metastases	17.7 ± 11.8	10.0 ± 8.6	0.001
ATA pediatric risk			0.089
Low	7 (19.4)	18 (30.5)	
Intermediate	6 (16.7)	17 (28.8)	
High	23 (63.9)	24 (40.7)	
Recurrence			
Yes	19 (52.8)	10 (16.9)	<0.001
No	17 (47.2)	49 (83.1)	

LNM, lymph node metastases; TSH, thyroid-stimulating hormone; Tg, thyroglobulin; CLND, central lymph node dissection; MRND, modified radical neck dissection; LND, lymph node dissection; ATA, American Thyroid Association.

### Analysis of the Risk Factors of Cervical Lymph Node Metastases in Children and Adolescents With Papillary Thyroid Carcinoma

As shown in **Table 3**, we analyzed the risk factors of LNM in children and adolescents with PTC and found that maximal tumor size >2 cm (p = 0.040) and T stage (p = 0.043) were the risk factors for central LNM. At the same time, our study showed that maximal tumor size >2 cm (p = 0.003), T stage (p = 0.005), number of central LNM (p < 0.001), and multifocality (p = 0.048) were the risk factors for lateral LNM. Multivariate analysis displayed that only the number of central LNM (OR 6.237, p = 0.005) was the independent risk factor for lateral LNM.

**Table 3:** Univariate and multivariate analyses of risk factors of cervical lymph node metastases.

Variables	Univariate		Multivariate		
	OR (95%)	p-Value	OR	95% CI	p-Value
**Central LNM**					
Maximal tumor size >2 cm	4.333 (1.071–17.539)	0.040			
T stage	2.647 (1.031–6.800)	0.043			
Multifocality	1.950 (0.550–6.915)	0.301			
Extrathyroidal extension	0.885 (0.217–3.614)	0.865			
Bilaterality	0.600 (0.168–2.138)	0.431			
**Lateral LNM**					
Maximal tumor size >2 cm	4.071 (1.615–10.262)	0.003			
T stage	2.763 (1.361–5.607)	0.005			
Central LNM	3.183 (0.885–11.447)	0.076			
Number of central LNM	8.482 (2.651–27.136)	<0.001			
Multifocality	2.462 (1.008–6.012)	0.048	6.237	1.747–22.266	0.005
Extrathyroidal extension	1.113 (0.430–2.879)	0.825			
Bilaterality	1.270 (0.500–3.228)	0.616			

OR, odds ratio; LNM, lymph node metastases.

We further analyzed the relationship between clinico-pathological features and the number of LNM. There was a significant difference in age at diagnosis, maximal tumor size >2 cm, T stage, and multifocality in the number of central LNM (p < 0.05). Our study also revealed that age at diagnosis, maximal tumor size >2 cm, T stage, the number of central LNM, and multifocality were the risk factors for the number of lateral LNM that approached statistical significance (p < 0.05). Moreover, multivariate analysis showed that multifocality (OR 3.575, p = 0.011; OR 3.175, p = 0.023) was the independent risk factor for the number of central and lateral LNM, respectively (**Table 4**).

**Table 4:** Factors related to the number of positive cervical lymph node metastasis (≤5 vs. >5).

Variables	Univariate			Multivariate		
	OR	95% CI	p-Value	OR	95% CI	p-Value
**Number of central LNM**						
Gender	0.433	0.181–1.039	0.061			
Age at diagnosis	0.326	0.138–0.771	0.011			
Maximal tumor size >2 cm	3.405	1.420–8.169	0.006			
T stage	3.193	1.563–6.524	0.001			
Multifocality	4.431	1.776–11.053	0.001	3.575	1.331–9.601	0.011
Extrathyroidal extension	1.331	0.542–3.266	0.533			
Bilaterality	1.572	0.672–3.681	0.297			
Preoperative TPOAb level	0.690	0.283–1.684	0.415			
Preoperative TgAb level	0.498	0.190–1.304	0.156			
Preoperative TSH level	1.528	0.557–4.191	0.410			
Preoperative Tg level	1.08	0.462–2.526	0.859			
**Number of lateral LNM**						
Gender	0.727	0.307–1.719	0.467			
Age at diagnosis	0.388	0.165–0.912	0.030			
Maximal tumor size >2 cm	6.167	2.480–15.331	<0.001			
T stage	3.558	1.724–7.340	0.001			
Central LNM	2.933	0.728–11.827	0.130			
Number of central LNM	6.517	2.639–16.092	<0.001			
Multifocality	4.208	1.739–10.183	0.001	3.175	1.171–8.608	0.023
Extrathyroidal extension	0.971	0.402–2.345	0.947			
Bilaterality	1.366	0.585–3.187	0.471			
Preoperative TPOAb level	0.933	0.390–2.236	0.877			
Preoperative TgAb level	0.742	0.296–1.859	0.524			
Preoperative TSH level	2.676	0.920–7.786	0.071			
Preoperative Tg level	1.994	0.844–4.713	0.116			

OR, odds ratio; LNM, lymph node metastases; TPOAb, thyroid peroxidase antibody; TgAb, thyroglobulin antibody; TSH, thyroid-stimulating hormone; Tg, thyroglobulin.

### Multivariate Analysis for Variables Associated With Papillary Thyroid Carcinoma Recurrence of Children and Adolescents

During a mean follow-up of 63 (2–193) months, 29 patients (30.5%) had a recurrence. The results of multivariate analysis for recurrence were summarized after adjusting for other clinicopathological factors, including age at diagnosis, gender, preoperative TPOAb, TgAb, TSH and Tg levels, multifocality, maximal tumor size, T stage, cervical LNM, N stage, and number of LNM. The results showed that age at diagnosis, positive preoperative TSH, maximal tumor size >2 cm, lateral LNM, number of LNM >5, N stage, and ATA pediatric risk were associated with poor prognosis in PTC patients with statistical significance (p < 0.05). Cox regression analysis found that younger age (<14 years) at diagnosis (HR 4.859, p = 0.001) and positive preoperative TSH (HR 2.416, p = 0.032) were independent risk factors for recurrence in children and adolescents with PTC (**Table 5**).

**Table 5:** Cox proportional hazards regression analysis for variables associated with PTC recurrence.

Variables	Univariate			Multivariate		
	HR	95% CI	p-Value	HR	95% CI	p-Value
<14 years old	4.150	1.924–8.953	<0.001	4.859	1.912–12.350	0.001
Positive preoperative TPOAb level	0.419	0.160–1.100	0.077			
Positive preoperative TgAb level	0.538	0.205–1.411	0.208			
Positive preoperative TSH level	2.961	1.389–6.314	0.005	2.416	1.077–5.420	0.032
Positive preoperative Tg level	2.068	0.994–4.301	0.052			
Maximal tumor size >2 cm	2.61	1.113–6.119	0.027			
T stage	1.691	0.985–2.905	0.057			
Number of central LNM>5	2.719	1.262–5.855	0.011			
Number of lateral LNM>5	5.031	2.044–12.388	<0.001			
Lateral LNM	4.322	1.306–14.309	0.017			
N stage	3.911	1.270–12.044	0.017			
Multifocality	1.982	0.878–4.477	0.100			
Extrathyroidal extension	0.950	0.418–2.158	0.903			
Bilaterality	0.556	0.237–1.302	0.176			
ATA pediatric risk	3.056	1.591–5.871	0.001			

HR, hazard ratio; TPOAb, thyroid peroxidase antibody; TgAb, thyroglobulin antibody; TSH, thyroid-stimulating hormone; Tg, thyroglobulin; LNM, lymph node metastases; ATA, American Thyroid Association.

The survival curves of DFS stratified by the presence of age at diagnosis, maximal tumor size, lateral LNM, the ATA pediatric risk stratification, preoperative TSH, and Tg are shown in **Figure 1**. The differences were significant in the age at diagnosis (p < 0.001, log-rank), maximal tumor size (p = 0.021, log-rank), lateral LNM (p = 0.009, log-rank), and ATA pediatric risk (p < 0.001, log-rank) in terms of the median DFS of patients with PTC (**Figures 1A–D**). The patients with positive preoperative TSH had a shorter median DFS (90.2 months) than the patients with negative preoperative TSH (136.0 months) (p = 0.003, log-rank) (**Figure 1E**). The median DFS was 116.4 months for patients with positive preoperative Tg and 119.1 months for patients with negative preoperative Tg (p = 0.046, log-rank) (**Figure 1F**).

## Discussion

Thyroid carcinoma is rare in children and adolescents, which accounts for 0.5%–3% (9), but the occurrence has been rising recently. Children and adolescents with PTC have unique biological characteristics; it has been proposed in the literature that they are more likely to have stronger invasiveness, higher LNM, and recurrence rate (10, 11). Therefore, it is necessary to do further research on the clinicopathological characteristics and prognosis of this age group to provide evidence for the clinical development of diagnosis and treatment plans. Thence, a central purpose of our study was to probe the relevant factors that influence the clinicopathological characteristics and prognosis significant in PTC of children and adolescents.

Previous studies have shown that PTC in children and adolescents has unique clinicopathological features. In order to explore the similarities and differences in clinicopathological features and prognosis of PTC between children and adolescents, we compared clinicopathological features between the younger group (<14 years) and the older group (14–18 years). Park et al. (12) showed that younger age was associated with more extensive forms of PTC, such as high ETE, multifocality, bilaterality, and maximal tumor size. Consistent with the results of Park, our study found that the cancer foci in the younger group were more aggressive and mainly multifocal, the preoperative TSH levels were often abnormal, the number of LN dissected and LN metastases were more than those of the older age groups, and the difference approached statistical significance (p < 0.05). Moreover, our study indicated that the recurrence rate of 52.8% in the younger age group was much higher than 16.9% in the older age group, similar to other research findings that children (<10 or <15 years) had a higher recurrence rate (13, 14). But others did not confirm this relationship (15). Therefore, further research is needed to confirm this conclusion.

The prevalence was reported in about 95% of neck LNM of PTC in children and adolescents (7, 8), and the presence of LNM has a significant impact on the recurrence rate (16, 17). However, due to the low incidence of PTC in children and adolescents and limited literature on the risk factors for LNM in this age group, it is necessary to do further study of the risk factors of LNM of PTC in children and adolescents. Our study found that maximal tumor size >2 cm and T stage were associated with central LNM (p < 0.05) and that maximal tumor size >2 cm, T stage, number of central LNM, and multifocality were the risk factors related to lateral LNM (p < 0.05), consistent with the report of the present study (18, 19). Moreover, multivariate analysis results showed that the number of central LNM (OR 6.237 p = 0.005) was an independent risk factor for lateral LNM, in line with other research findings (16). Our study displayed that ETE was not an independent risk factor for lateral LNM, which may be because the association between ETE and LNM was based on tumor size. Our research results demonstrated that the lateral LNM of PTC was also mainly related to factors delegating tumor aggressiveness and progression in children and adolescents.

The above results showed that the number of central LNM was an independent risk factor for lateral LNM. In order to explore the related factors that affect the number of LNM of PTC in children and adolescents, we divided the number of LNM into two groups (≤5 and>5). Univariate analysis results displayed that age at diagnosis, maximal tumor size >2 cm, T stage, and multifocality were the risk factors of the number of central and lateral LNM (p < 0.05). Moreover, central LNM and the number of central LNM were also the risk factors of the number of lateral LNM (p < 0.05). Multivariate analysis results showed that only multifocality (OR 3.175 p = 0.023) was an independent risk factor for the number of lateral LNM. Therefore, patients with the above characteristics may consider prophylactic LN dissection.

The recurrence rate of PTC in children and adolescents is high, and the second operation for recurrence has a greater impact on this age group. Because of an increasing incidence of PTC in this age group, documentation of the prognosis and identification of predictors of DFS are of great clinical value. Our results showed that age at diagnosis, positive preoperative TSH level, maximal tumor size >2 cm, number of central and lateral LNM, lateral LNM, and N staging were risk factors for recurrence, similar to other research findings (20–22). In addition, we also found that the ATA pediatric risk stratification had predictive value for recurrent PTC patients in children and adolescents, the same results as other scholars’ research (22, 23). Multivariate analysis demonstrated that younger age (<14 years) at diagnosis and positive preoperative TSH were independent risk factors for recurrence, which were consistent with the results of other studies (20–25). The prognosis of pediatric PTC is generally excellent. However, factors such as younger age, positive preoperative TSH levels, maximal tumor size >2 cm, lateral LNM, and number of central and lateral LNM are more prone for disease recurrence and should always be considered in the management of these patients.

The related research suggested that, in children, prophylactic central neck dissection (CND) was associated with increased DFS, as high as 95% at 5 and 10 years (26). Another study showed that prophylactic CND may reduce the risk for reoperation that was as high as 77% in those without CND (27). Some researchers advised that high-volume surgeons can carry out a safe total thyroidectomy (TT) or non-total thyroidectomy (NTT) and routine central CND (28). Both the CAEK and ATA recommended that on pediatric thyroid cancer for patients with PTC and no clinical evidence of gross extrathyroidal invasion and/or locoregional metastasis, prophylactic CND may be selectively considered based upon tumor focality, size, and the experience of the surgeon (29, 30). Our study suggested that patients with features of risk factors for recurrence such as younger age, positive preoperative TSH, maximal tumor size >2 cm, lateral LNM, and number of LNM >5 may be considered prophylactic or therapeutic dissection of additional metastatic LNs by high-volume surgeons to prevent and reduce the recurrence rate of patients during long-term follow-up.

There are some limitations in our study: 1) this is a single-center retrospective study; and 2) pathological subtypes and BRAF mutation status were not considered in this study. These limitations need to be improved in the future.

## Conclusions

In our study cohort, the clinicopathological characteristics of younger age (<14 years) were highly aggressive and prone to metastases, the preoperative TSH were mostly abnormal, and the recurrence rate was much higher than that of older age (14–21 years). Tumor size and T stage were risk factors for neck LNM; central LNM, the number of central LNM, and multifocality were risk factors for lateral LNM. Moreover, multivariate analysis showed the number of central LNM was an independent risk factor for lateral LNM. Univariate analysis results showed that younger age (<14 years) at diagnosis, positive preoperative TSH, maximal tumor size >2 cm, lateral LNM, number of LNM, N staging, and ATA pediatric risk were associated with poor prognosis of PTC in children and adolescents. Cox regression analysis found that younger age (<14 years) at diagnosis and positive preoperative TSH were independent risk factors for recurrence of PTC in children and adolescents. Therefore, patients with characteristics such as younger age (<14 years) at diagnosis, positive preoperative TSH, maximal tumor size >2 cm, lateral LNM, and number of LNM >5 may be considered for prophylactic or therapeutic dissection of additional metastatic LNs by high-volume surgeons to prevent and reduce the recurrence rate of patients during long-term follow-up.

## Data Availability Statement

The raw data supporting the conclusions of this article will be made available by the authors, without undue reservation.

## Ethics Statement

The studies involving human participants were reviewed and approved by the Ethical Committee of the Tianjin Medical University Cancer Institute and Hospital. The patients provided written informed consent to participate in this study.

## Author Contributions

YG and DH: conceptualization, data collection and analysis, methodology, and drafting of the manuscript. YH: conceptualization, data collection and analysis, and methodology. XW and JZ: conceptualization and methodology. JW: conceptualization and manuscript review and editing. All authors listed have made a substantial, direct, and intellectual contribution to the work and approved it for publication.

## Funding

This work was supported by grants from the Natural Science Foundation of Tianjin City (18JCYBJC92900).

## Conflict of Interest

The authors declare that the research was conducted in the absence of any commercial or financial relationships that could be construed as a potential conflict of interest.

## Publisher’s Note

All claims expressed in this article are solely those of the authors and do not necessarily represent those of their affiliated organizations, or those of the publisher, the editors and the reviewers. Any product that may be evaluated in this article, or claim that may be made by its manufacturer, is not guaranteed or endorsed by the publisher.

## Abbreviations

PTC, papillary thyroid carcinoma; ETE, extrathyroidal extension; ATA, American Thyroid Association; DFS, disease-free survival; TSH, thyroid-stimulating hormone; Tg, thyroglobulin; TPOAb, thyroid peroxidase antibody; TgAb, thyroglobulin antibody; LNM, lymph node metastases; LN, lymph node; LND, lymph node dissection; CLND, central lymph node dissection; MRND, modified radical neck dissection; HR, hazard ratio; OR, odds ratio; CND, central neck dissection; CAEK, guidelines from the German Association of Endocrine Surgeons; TT, total thyroidectomy; NTT, non-total thyroidectomy.
